# Does social disadvantage over the life-course account for alcohol and tobacco use in Irish people? Birth cohort study

**DOI:** 10.1093/eurpub/ckt122

**Published:** 2013-09-10

**Authors:** Jayati Das-Munshi, Gerard Leavey, Stephen A. Stansfeld, Martin J. Prince

**Affiliations:** 1 Department of Health Service & Population Research, Institute of Psychiatry, King’s College London, London, UK; 2 Bamford Centre for Mental Health & Well-Being, University of Ulster, Derry-Londonderry, UK; 3 Centre for Psychiatry, Queen Mary University of London, London, UK

## Abstract

**Aims:** Few studies have examined how the settlement experiences of migrant parents might impact on the downstream adult health of second-generation minority ethnic children. We used prospective data to establish if childhood adversity relating to the settlement experiences of Irish-born parents might account for downstream adverse health-related behaviours in second-generation Irish respondents in adulthood. **Design, setting and participants:** Cohort data from the National Child Development Study, comprising 17 000 births from a single week in 1958, from Britain, were analysed. Respondents were followed to mid-life. Dependent variables were alcohol and tobacco use. The contribution of life-course experiences in accounting for health-related behaviours was examined. **Findings:** Relative to the rest of the cohort, the prevalence of harmful/hazardous alcohol use was elevated in early adulthood for second-generation men and women, although it reduced by age 42. Second-generation Irish men were more likely to report binge alcohol use (odds ratio 1.45; 95% confidence interval 0.99, 2.11; *P* = 0.05), and second-generation Irish women were more likely to smoke (odds ratio 1.67; 95% confidence interval 1.23, 2.23; *P* = 0.001), at mid-life. Childhood disadvantage partially mediated associations between second-generation Irish status and mid-life alcohol and tobacco use, although these were modest for associations with smoking in Irish women. **Conclusions:** The findings suggest mechanisms for the intergenerational ‘transmission’ of health disadvantage in migrant groups, across generations. More attention needs to focus on the public health legacy of inequalities transferring from one migrant generation to the next.

## Introduction

The ethnic minority population in Britain stood at 9.1 million from 2001 to 2009, of which a sizeable proportion reflected a growing British-born ethnic minority population.^1^ Much of the literature on health inequalities in minority ethnic groups has focused on first-generation migrants, with scant research on the health of second/later-generation minority ethnic groups. Whereas research on migrant health has suggested ‘healthy migrant’ selection effects,^2^ second-generation ethnic minority groups appear to have poorer health and health-related behaviours, despite improvements in socioeconomic position.^3^ Using a life-course framework to examine ethnic health inequalities allows an examination of the contribution of childhood to adulthood experiences in the patterning of downstream adverse health outcomes and/or health-related behaviours.

Irish people living in Britain, of either first-, second- or third-generation Irish descent, constitute 11% of the population (approximately 6 million people).^4^ Despite this, the health needs of Irish people have been relatively neglected.^4^ Irish people living in Britain experience elevated mortality^5^ and physical as well as psychological morbidity,^6–8^ relative to the rest of the population. This has been noted in the first generation and has persisted into the second, despite improvements in socioeconomic position.^5^^,^^6^^,^^8^

There has been debate over whether this might be the result of adverse health-related behaviours such as alcohol or tobacco use.^9^^,^^10^ However, there have been few studies specifically examining alcohol and tobacco use in second-generation Irish people.^11^ The available evidence suggests that the prevalence of alcohol and tobacco use in people of second and later Irish descent is similar to the general population in Britain.^3^^,^^10^ Such studies are limited by the use of single time-points in cross-sectional analyses.

We analysed data from a prospective nationally representative birth cohort from Britain, namely, the National Child Development Study (NCDS).^12^ Our objectives were to assess patterns of alcohol and tobacco use and social disadvantage over the life-course in second-generation Irish people growing up in Britain. We hypothesized that social disadvantage experienced over the life-course would account for observed differences in tobacco and alcohol use at mid-life, among second-generation Irish respondents.

## Methods

### Study sample

In all, 18 558 babies born during 3–9 March 1958 (98% of live births) were identified for the study. Of this, 920 children were excluded, as they were born outside Britain. A further 873 children were excluded, as one or both parents were born outside Britain or Ireland. This resulted in 16 765 children. Parents, teachers and medical personnel were interviewed when children were 7, 11 and 16 years old. At age 23, 33, 42 and 44/45, cohort members were interviewed. The ‘target sample’ was restricted to children born in England, Scotland and Wales, and children with both parents born in these countries or with one/both parents born in Ireland or Northern Ireland. Parents reported their country of birth when cohort members were 11 and 16 years old. If either parent reported being born in Ireland or Northern Ireland, cohort members were classified as ‘second generation Irish’. Excluding non-responders, kappa assessing the reliability of parental responses to this question between the two sweeps was high (kappa = 0.97).

### Measures

#### Material and social adversity measures

Adversity indicators assessed in childhood were household overcrowding, access to household amenities (hot water, indoor toilet and bathroom) and whether cohort members received free school meals. In addition, health visitors noted any ‘family difficulties’ (problems with housing, finances, physical illness/disability, mental illness, learning disabilities, death of parent(s), divorce/separation, domestic tension, in-law conflicts, unemployment, alcoholism or ‘other serious difficulties affecting child’s development’). This was analysed as a binary measure (‘no family difficulties’ vs. ‘one or more family difficulties’). Parents were asked if they smoked when children were 16. Respondents were asked at age 44/45 if they had experienced financial hardship and if either parent had alcohol or emotional problems, in their childhood. In adulthood, respondents were asked about if they had experienced periods of unemployment, homelessness, or if they were in receipt of benefits or residence in social housing; whether they had financial difficulties/difficulties paying for bills, food or clothing and whether they had access to a car, telephone, central heating and an indoor toilet/bathroom or damp in their house. Social support was assessed at age 33. At age 44/45, social support was assessed with the Close Person’s Questionnaire.^13^ Marital status at age 23, 33 and 42 was noted. Cohort members reported stressful life events in the previous 6 months and were asked about perceived job security at age 44/45.

#### Psychological health

At age 7 and 11, teachers rated children’s emotional health using the Bristol Social Adjustment Guide.^14^ At 16, the teacher-rated Rutter Behavioural Scale was used.^15^ ‘Internalising’ problems comprised worries, solitary, miserable, fearful and fussy.^16^ ‘Externalising’ problems comprised fights, destructive, not liked by other children, lies, steals, resentful, irritable, disobedient and bullies other children.^16^ Scores were summed and square root transformed; the top 13% indicated a ‘case’.^16^ At age 23 and 33, cohort members completed the Malaise Inventory, scores of ≥8 indicating psychological distress.^6^ An abbreviated version of the *Clinical Interview Schedule-Revised*^17^ was used to assess common mental disorders (CMDs) at age 44/45. Cut-off points of 8/9 indicated CMD on this measure.^6^

#### Health-related behaviours

At age 16, respondents were asked if they had tried alcohol. At age 33 and 42, respondents were asked questions from the CAGE (‘Have you wanted to **C**ut down your alcohol use lately?’ ‘Do you get **A**ngry if other people suggest you should cut down your alcohol use?’ ‘Do you feel **G**uilty about the amount of alcohol you consume?’ ‘Have you ever needed an **E**ye-opener?’).^18^ Cut-offs of 1+ indicated hazardous alcohol use. This cut-off has greater sensitivity for alcohol misuse and dependency than cut-offs of 2+.^19^ At age 23, 33 and 42, respondents reported amounts of alcohol consumed within the previous week; drinking >50 U/week indicated harmful use. At age 44/45, the Alcohol Use Disorders Identification Test (AUDIT) assessed alcohol misuse; scores of 8+ indicated hazardous alcohol use.^20^ At age 44/45, respondents reported if they had drunk ‘six or more (standard) drinks’ in one sitting in the previous month, indicating ‘binge alcohol use’. At age 23, 33 and 42, cohort members reported if they abstained from alcohol use. Cohort members reported if they were current, previous or ‘never smokers’ at age 23, 33 and 42.

### Statistical analysis

STATA 12^21^ was used for analyses. All models were gender stratified. Gender interactions were assessed for statistical significance. Multivariate logistic regression analysis assessed the association of ‘parental migration history’ (i.e. second-generation Irish cohort members vs. cohort members without a parental history of migration) with the dependent variables of alcohol or tobacco use behaviours.

To assess for the contribution of variables in mediating excess risks of adverse alcohol and tobacco behaviours at mid-life in second-generation Irish cohort members relative to the rest of the cohort, three criteria needed to be fulfilled.^22^ First, the association of parental migration history with putative mediator had to be established. Second, the association of the putative mediator with the dependent variable was assessed. Finally, the association of parental migration history with the dependent variable was assessed after adjusting for the putative mediator. If the coefficient for the association between parental migration history and the dependent variable was reduced in the presence of the putative mediator, then the data were considered supportive of mediation.^22^ Wald tests were used to assess strengths of associations and gender interactions.

#### Missing data

As missing values were likely to be missing at random, missing data were imputed using the chained equations approach (‘*ICE*’) in STATA.^23^ Imputations were conducted on all cohort members alive at the time of the biomedical survey, at age 44/45. Fifty imputed datasets were created using proper imputation from an imputation model in which all covariates in the model as well as additional variables known to predict attrition (mother’s education, region of birth, employment at 33 and social class at all sweeps) were included.^24^

## Results

At age 7, 11, 16, 23, 33 and 42, response rates were 89, 88, 84, 72, 65 and 66%, respectively.^12^ Within the target sample, complete data were available for the AUDIT for 8671 individuals (92% of the biomedical sample), and on smoking in 9079 individuals (97% of the biomedical sample). Response rates and reasons for non-response of Irish cohort members were similar to the rest of the sample (see Supplementary table S1).

Table 1 displays life-course alcohol use in second-generation Irish cohort members. Compared with the rest of the cohort, second-generation Irish men had increased relative odds of harmful levels of alcohol use in early adulthood, which diminished by mid-life. Both second-generation Irish men and women were more likely to report hazardous alcohol use relative to the rest of the cohort at age 33, although this risk diminished by mid-life. At all time points, second-generation Irish women were more likely to report that they abstained from alcohol relative to other women in the cohort, although gender interactions were only significant for this outcome at age 42 (table 1). At age 44/45, there were few differences between Irish men and women and the rest of the cohort on AUDIT cut-offs, although second-generation Irish men were more likely to report that they had binge drunk within the previous month relative to men in the rest of the cohort [odds ratio (OR) 1.45; 95% confidence interval (CI) 0.99, 2.11; *P* = 0.05] (table 1).

**Table 1:** Alcohol use in second-generation Irish cohort members relative to the rest of the cohort: Univariate analyses

Total in sample	Age	Year		Second-generation Irish men^a^		Second-generation Irish women^b^	
*N*				OR	(95% CI)	OR	(95% CI)
Tried alcohol							
9175	16	1974	Tried alcohol vs. not	0.95	0.52, 1.74	0.65	0.43, 0.98
Harmful alcohol use across the life-course							
9436	23	1981	Drank >50 U in last week	1.42	1.03, 1.96	1.15	0.27, 4.85
8877	42	2000	Drank >50 U in last week	1.13	0.77, 1.65	1.06	0.33, 3.40
Hazardous alcohol use across the life-course							
10 095	33	1991	≥1 on CAGE	1.44	1.12, 1.86	1.63	1.24, 2.15
10 091	42	2000	≥1 on CAGE	1.23	0.94, 1.59	1.05	0.78, 1.40
Abstained from alcohol							
11 068	23	1981	Abstained	0.87	0.40, 1.88	1.24	0.75, 2.05
10 135	33	1991	Abstained	0.51	0.16, 1.59	1.38	0.84, 2.25
10 210	42	2000	Abstained	0.28	0.07, 1.08	1.22	0.75, 1.99*
Mid-life (age 44/45) drinking behaviours							
7882	44/45	2002	Binge alcohol use	1.45	0.99, 2.11	1.15	0.86, 1.54
7877	44/45	2002	≥8 on the AUDIT	1.09	0.82, 1.45	1.24	0.86, 1.80

a: Relative to non-Irish men in cohort.

b: Relative to non-Irish women in the cohort.

*Statistically significant interactions with gender and ethnicity were noted for this outcome (*P* < 0.05).

**Figure 1:**
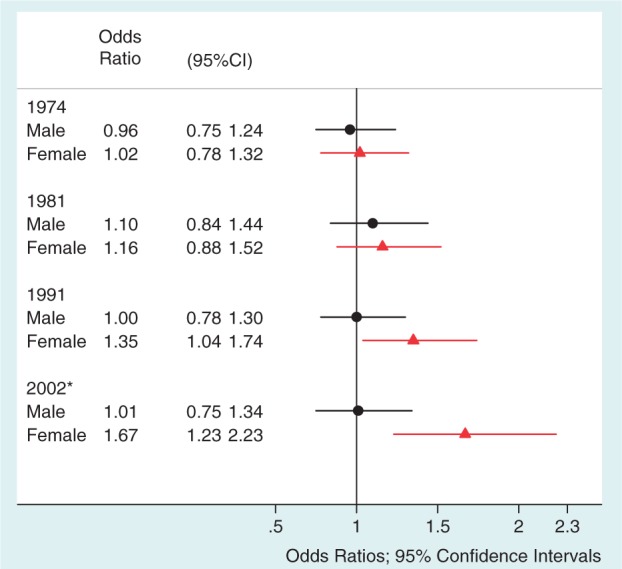
Plot of relative odds of being a smoker/ex-smoker vs. non-smoker in second-generation Irish men and women relative to men and women in the rest of the cohort. Circles indicate ORs in second-generation Irish men relative to men in the non-Irish reference group; Triangles indicate ORs in second-generation Irish women relative to women in the non-Irish reference group. Horizontal lines indicate 95% CIs. Estimates falling on the vertical line indicate no difference between second-generation Irish participants and the non-Irish reference group. *P value for interaction of gender with ethnicity: *P* = 0.02

Whereas second-generation Irish men had similar patterns of smoking across all time points compared with men in the reference population, second-generation Irish women had an elevated odds of cigarette smoking, relative to women in the rest of the cohort, which increased by mid-life (figure 1). Taking all adult time points together (age 23, 33 and 42), second-generation Irish women were 1.83 times more likely than women in the rest of the cohort to report being a smoker at least once (95% CI 1.14, 2.93; *P* = 0.01), whereas the odds ratio was 1.29 (95% CI 0.83, 2.02; *P* = 0.26) in second-generation Irish men.

Relative to the rest of the cohort, second-generation Irish cohort members were more likely to grow up in marked material and social hardship (Supplementary table S2). By mid-life, second-generation Irish cohort members had a similar profile of adversity to the rest of the cohort, suggesting that a large amount of differential upward social mobility had taken place (Supplementary table S2).

Supplementary table S3 displays gender-adjusted associations of adverse experiences over the life-course, with the mid-life outcomes of binge alcohol use, hazardous alcohol use and smoking. Strong associations for most adverse experiences over the life-course with these dependent variables were noted in the full sample.

The role of life-course experiences in mediating differences between second-generation Irish cohort members and the rest of the cohort is displayed in table 2. As differences were only noted for mid-life binge alcohol and tobacco use, only these were assessed for mediation. Across most models, childhood adversity partially mediated the association between second-generation Irish status and mid-life binge alcohol use and smoking. This was more modest for mid-life smoking in second-generation Irish women, in whom the relative odds remained elevated at 1.50 (95% CI 1.10, 2.05), even after all putative mediators had been added into the model.

**Table 2:** Which factors over the life-course mediate differences in tobacco and alcohol misuse in second-generation Irish cohort members, relative to the rest of the cohort?

Model		Second-generation Irish (men and women)^a^		Second-generation Irish men^b^		Second-generation Irish women^b^	
		OR	(95% CI)	OR	(95% CI)	OR	(95% CI)
Smoking at age 44/45; *n* = 8220							
1	Baseline model	1.29	1.05, 1.58	1.01	0.75, 1.34	1.67	1.24, 2.24
2	Mid-life	1.30	1.06, 1.60	1.03	0.77, 1.38	1.66	1.22, 2.24
3	Early adulthood	1.24	1.00, 1.53	1.00	0.74, 1.35	1.56	1.14, 2.12
4	Childhood	1.18	0.95, 1.47	0.94	0.69, 1.27	1.50	1.10, 2.05
Binge alcohol use at age 44/45; *n* = 7882							
1	Baseline model	1.26	1.00, 1.58	1.45	0.99, 2.11	1.15	0.86, 1.54
2	Mid-life	1.24	0.98, 1.56	1.46	1.00, 2.14	1.11	0.83, 1.49
3	Early adulthood	1.20	0.95, 1.51	1.42	0.97, 2.08	1.07	0.81, 1.45
4	Childhood	1.14	0.90, 1.44	1.34	0.91, 1.97	1.02	0.75, 1.39

a: Relative to men and women in the rest of the cohort.

b: Gender-stratified analyses; relative to Irish men or women in the rest of the cohort. Model 1: Adjusted for gender; Model 2: Adjusted for gender + mid-life: material adversity, social support, marital status, stressful life events, job security and common mental disorders; Model 3: Adjusted for variables in models 1, 2 + early-adulthood: material adversity, social support, marital status, psychological health; Model 4: Adjusted for variables in models 1, 2, 3 + childhood: material adversity, parental emotional problems/health-related behaviours, family difficulties and psychological problems. The full list of variables is displayed in Supplementary tables S2 and S3.

## Discussion

### Main findings and implications

The issue of alcohol misuse, dependency and tobacco use in the Irish community may be contentious. For some, it is an unwelcome labelling of Irish people that obfuscates the wider agenda on health inequalities with potential detriment to policy; it has been suggested that the implication of (focusing on health-related behaviours) is that ‘*the Irish in England do not behave and they pay for it by death*’.^9^ Attributing all health differences to health-related behaviours may miss other causes for health inequalities in Irish people.^25^ However, to avoid this topic because of concerns over stereotyping may also be of a disservice to Irish communities in Britain.^11^ For example, whereas there has been a decline in tobacco use in the general population over the past decade, similar reductions have not been seen among ethnic minority groups living in Britain^26^; this has important implications for addressing health inequalities.

This study revealed complex patterns of tobacco and alcohol use over the life-course in second-generation Irish men and women. Second-generation Irish men and women were more likely than the rest of the cohort to report harmful or hazardous alcohol behaviours in early adulthood, but this was reduced by mid-life. However, we found an elevated prevalence of reported binge alcohol use at mid-life among second-generation Irish men relative to their contemporaries. High levels of abstention from alcohol were also noted among second-generation Irish women, consistent with a previous study suggesting high levels of abstention among Irish people living in Britain mixed with a picture of greater use among those who reported any alcohol use at all.^10^

The odds of reported tobacco use among second-generation Irish women relative to women in the rest of the cohort increased from 1991 to 2001; this trend was not seen for Irish men. This might indicate that wider public health policy around smoking has failed to reach this group, a concern that has previously been raised for other ethnic minority groups.^26^

Irish respondents were more likely than the rest of the cohort to grow up under circumstances of marked disadvantage. By adulthood, parity on most adversity measures had been reached. This is consistent with evidence on intergenerational social class mobility among ethnic minority communities in Britain.^27^ For example, second-generation Irish people attain educational qualifications equivalent to, or better than, white British counterparts,^4^ which has led commentators to consider the role of Catholic schools in enhancing educational attainment and social mobility in Irish children.^4^

The findings suggest that childhood disadvantage and parental health-related behaviours exert long-range effects on mid-life health-related behaviours in second-generation Irish cohort members. The role of childhood adversity in accounting for later health-related behaviours may indicate a ‘sensitive period’ during which intervention could lead to improvements in downstream adult health outcomes. This is consistent with the wider literature, where it has been suggested that childhood experiences contribute to later adult alcohol and tobacco behaviours.^28^

### Strengths and Limitations

The data derived from a sample of births from a single week in 1958, and so can be assumed to be nationally representative. Most measures were prospective; this minimized the possibility of measurement error. Recalled measures assessed sensitive topics such as parental alcohol misuse; although these are prone to recall bias, there is no reason to believe that this would have been differential with respect to ethnicity.

An assumption of mediation analysis is that all putative mediators have been specified and accurately assessed.^22^ It is possible that there were other variables that we did not have information on, or that measurement was not perfect. Given these limitations, our findings on mediation should be regarded as tentative, although they are consistent with the wider literature.^6^^,^^29^

As this was a historical cohort analyses, data relating specifically to the experiences of being the child of migrant parents in 1958 were not available. Therefore, other important migration-related experiences such as neighbourhood context,^30^ pre-migratory experiences of Irish-born parents^7^ and experiences of discrimination^7^ could not be examined. It is likely that period effects, for example relating to tobacco policy, might also have impacted on the findings, although this would have been non-differential with respect to ethnicity. Also, other period effects could not be assessed in this historical cohort. For example, we were unable to directly assess the effect of heightened political tensions in Northern Ireland in 1981 (when respondents would have been aged 23), on cohort members’ health. It is possible that Irish respondents experienced anti-Irish discrimination over this period, which may have impacted on health-related behaviours.^31^

### Unanswered questions and future research

The findings from the present study relate to a very specific period in British history, in which migration from Ireland to Britain saw an increase, partly facilitated by the Ireland Act of 1949, which allowed Irish citizens to settle in Britain with relatively little restriction.^32^ Irish-born migrants to Britain at this time represented a ‘first wave’, many of whom arrived to take up work in the poorly paid domestic sector and construction industries.^32^ This feature of Irish settlement in Britain in the 1940s/50s may account for the relative material disadvantage that second-generation Irish children were born into in this study. Although a concern is that the findings may only be specific to the period in which this cohort was conceived, analyses using data from the 1970 British Birth Cohort has also confirmed high levels of material disadvantage experienced in childhood among second-generation Irish children, alongside poorer mental health in Irish-born parents,^33^ suggesting consistency across time. Analyses from more recent birth cohorts may help to shed light on whether the phenomena described in this article are relevant to other second-generation ethnic minority groups, who may be born into poverty, but who may then experience high levels of upward intergenerational social mobility.^3^^,^^27^^,^^34^ Future research should examine health inequalities in migrant and ethnic minority groups across the full life-course.^34^

### Policy implications

Previous research and policy has tended to focus on Irish-born migrants.^11^ The present study lends credence to the observation that these health inequalities are not just limited to Irish-born migrants, but continue to be felt by the second generation. The findings highlight the long-range impact of childhood adversity on mid-life health-related behaviours. This has also been demonstrated for mid-life CMDs and well-being, in this cohort.^6^ The non-specificity of childhood adversity in accounting for a number of later adult health outcomes is a powerful observation, as intervening at earlier stages in the life-cycle could have far-reaching positive effects on a variety of health outcomes.

## Supplementary Data

Supplementary data are available at *EURPUB* online.

## Funding

Medical Research Council (MRC) training fellowship awarded to J.D-M; Grant number: G0701595/1. The funder did not play any part in design of the research protocol, data analysis or writing of the report. The analyses in this work are based wholly on analysis of data from the National Child Development Study (NCDS). The data were deposited at the UK Data Archive by the Centre for Longitudinal Studies at the Institute of Education, University of London. NCDS is funded by the Economic and Social Research Council (ESRC).

*Conflicts of interest*: None declared.

**Key points**

- Ethnic minority groups continue to experience poorer health despite improvements in socioeconomic position across generations.
- Intergenerational health inequalities are marked in Irish-descended people, who experience elevated mortality despite improvements in socioeconomic position across generations.
- Few studies have used prospective cohort data to assess how the settlement experiences of migrant families may negatively impact on the adult health of second-generation children.
- Using nationally representative birth cohort data, the present study tracked the lives of second-generation Irish people growing up in Britain. By mid-life, Irish men were more likely to report binge alcohol use and Irish women were more likely to report smoking, relative to the rest of the cohort.
- Childhood disadvantage and parental health-related behaviours partially accounted for alcohol and tobacco behaviours at mid-life in Irish cohort members, suggesting that health disparities in adulthood may have social origins in childhood.

## Supplementary Material

Supplementary Data
