# Supplementary material for: Does social disadvantage over the life-course account for alcohol and tobacco use in Irish people? Birth cohort study
Source: Eur J Public Health. 2013 Sep 10;24(4):594–9. doi: 10.1093/eurpub/ckt122 (PMC4110955; doi:10.1093/eurpub/ckt122)
Supplement: Supplementary Data [file supp_24_4_594__index.html]

Does social disadvantage over the life-course account for alcohol and tobacco use in Irish people? Birth cohort study — Does social disadvantage over the life-course account for alcohol and tobacco use in Irish people? Birth cohort study — Supplementary Data 

# Does social disadvantage over the life-course account for alcohol and tobacco use in Irish people? Birth cohort study

## Supplementary Data

files

**Files in this Data Supplement:**

- Supplementary Data - docx file
