# Supplementary material for: Does social disadvantage over the life-course account for alcohol and tobacco use in Irish people? Birth cohort study
Source: Eur J Public Health. 2013 Sep 10;24(4):594–9. doi: 10.1093/eurpub/ckt122 (PMC4110955; doi:10.1093/eurpub/ckt122)
Supplement: Supplementary Data [file supp_ckt122_ejph-2012-10-om-0812-File003.docx]

**ONLINE REPOSITORY MATERIAL**

**Supplementary table 1: Response rates at each sweep of NCDS (un-imputed data)**

| **Sweep (age- years)** | **0 (0)** | **1 (7)** | **2 (11)** | **3 (16)** | **4 (23)** | **5 (33)** | **6 (42)** | **Biomedical sweep (44/45)** |
| --- | --- | --- | --- | --- | --- | --- | --- | --- |
| **Year** | 1958 | 1965 | 1969 | 1974 | 1981 | 1991 | 2000 | 2002 |
| **Number (% of total (n=16765*)) present in analysis sample at each sweep** | 16553 (99%) | 14258 (85%) | 13915 (83%) | 13138 (78%) | 11411 (68%) | 10460 (62%) | 10412 (62%) | 8690 (52%) |
| **Number (% of total (n=791**)) of second generation Irish respondents in analysis sample** | 782 (99%) | 710 (90%) | 761 (96%) | 699 (88%) | 544 (69%) | 509 (64%) | 505 (64%) | 417 (53%) |

***Key****:*Excludes children who migrated to Britain and were not born in England, Scotland or Wales in the index week, 1958 (n=920).* *Also excludes children who had one or both parents born outside England, Scotland, Wales, Ireland or Northern Ireland (n=1251); **After excluding migrant children, there were 791 children who were second generation Irish within NCDS. Top row of figures includes Irish respondents in the totals.*

**ONLINE RESPOSITORY MATERIAL**

| **Supplementary Table 2**  **Univariate associations of parental migration history with material and social adversity indicators over the life-course.**  ***Odds ratios and 95% Confidence Intervals compare experiences in second generation Irish cohort members to the rest of the cohort*.** | | | | | | | | | |
| --- | --- | --- | --- | --- | --- | --- | --- | --- | --- |
|  | |  | ***Men*** | |  |  | ***Women*** | |  |
| **Variable, age** | | **N** | **OR** | **(95% CI)** | **p** |  | **OR** | **(95% CI)** | **p** |
| ***CHILDHOOD (AGE 7, 11, 16)*** | | | | | | | | | |
| **Material and social adversity indicators** | | | | | | | | | |
| Household overcrowding∞ | | 7963 | 2.30 | 1.69,3.12 | p<0.001 |  | 2.25 | 1.62, 3.10 | p<0.001 |
| Restricted access to basic household amenities^1^ | | 8085 | 1.54 | 1.13,2.09 | 0.01 |  | 1.56 | 1.12, 2.15 | p<0.001 |
| Family financial difficulties | | 7091 | 2.25 | 1.62,3.12 | p<0.001 |  | 2.21 | 1.57, 3.11 | p<0.001 |
| Family difficulties^2^assessed by health visitor, age 7 | | 9816 | 2.21 | 1.67,2.94 | p<0.001 |  | 1.54 | 1.14, 2.08 | 0.005 |
| Grew up in poverty or financial hardship** | | 8019 | 1.50 | 1.07,2.11 | 0.02 |  | 2.14 | 1.55, 2.96 | p<0.001 |
| **Parental mental health and health-related behaviours** | | | | | | | | | |
| Either parent smoked, age 16 | | 9133 | 1.38 | 1.01,1.88 | 0.04 |  | 1.29 | 0.93, 1.78 | 0.13 |
| Either parent had alcohol problems** | | 8161 | 2.17 | 1.53,3.08 | p<0.001 |  | 2.12 | 1.53, 2.93 | p<0.001 |
| Either parent suffered from emotional/ nervous trouble** | | 8159 | 1.14 | 0.82,1.59 | 0.43 |  | 1.41 | 1.05, 1.91 | 0.02 |
| **Cohort member psychological health (childhood)** | | | | | | | | | |
| Childhood internalising problems at least once | | 8704 | 1.20 | 0.90,1.59 | 0.21 |  | 1.22 | 0.90, 1.67 | 0.20 |
| Childhood externalising problems at least once | | 8619 | 1.30 | 0.98,1.72 | 0.07 |  | 1.15 | 0.81, 1.64 | 0.43 |
| ***ADULTHOOD (AGE 23, 33, 42, 44/ 45)*** | | | | | | | | | |
| **Material and social adversity indicators** | | | | | | | | | |
| **Age 23** |  | |  |  |  |  |  |  |  |
| Unemployed | 11047 | | 1.04 | 0.71,1.52 | 0.83 |  | 1.48 | 0.97, 2.26 | 0.07 |
| In receipt of benefits | 11058 | | 1.35 | 1.05,1.74 | 0.02 |  | 1.08 | 0.84, 1.39 | 0.55 |
| Homeless since last sweep | 11057 | | 0.96 | 0.57,1.60 | 0.86 |  | 1.39 | 0.87 2.22 | 0.17 |
| Lives in council housing | 10758 | | 1.24 | 0.87,1.76 | 0.24 |  | 1.21 | 0.89, 1.66 | 0.23 |
| Shared/ no use of indoor bath or shower | 10763 | | 0.92 | 0.48,1.73 | 0.79 |  | 1.09 | 0.57, 2.10 | 0.79 |
| Shared/ no access to indoor toilet | 10763 | | 0.92 | 0.53,1.59 | 0.76 |  | 1.13 | 0.66, 1.93 | 0.65 |
| Household overcrowding∞ | 10493 | | 1.55 | 0.89,2.71 | 0.12 |  | 1.36 | 0.68, 2.70 | 0.38 |
| **Age 33** |  | |  |  |  |  |  |  |  |
| Qualifications (higher vs. none) | 9290 | | 1.40 | 0.87,2.24 | 0.16 |  | 0.79 | 0.55, 1.12 | 0.19‡ |
| Lack of central heating in home | 10224 | | 1.21 | 0.83,1.77 | 0.31 |  | 0.75 | 0.54, 1.03 | 0.08 |
| Resident in social housing | 9290 | | 1.22 | 0.83,1.79 | 0.30 |  | 0.87 | 0.61, 1.25 | 0.45 |
| Damp in home | 10068 | | 1.08 | 0.74,1.59 | 0.68 |  | 1.17 | 0.82, 1.68 | 0.39 |
| No access to telephone | 10044 | | 0.96 | 0.59,1.56 | 0.88 |  | 1.00 | 0.61, 1.63 | 1.00 |
| In arrears with bills | 10224 | | 1.06 | 0.49,2.30 | 0.88 |  | 1.68 | 0.92, 3.08 | 0.09 |
| Household overcrowding∞ | 10045 | | 1.25 | 0.85,1.83 | 0.26 |  | 0.95 | 0.65, 1.39 | 0.78 |
| Unemployed | 10136 | | 1.06 | 0.66,1.69 | 0.81 |  | 0.69 | 0.28, 1.68 | 0.41 |
| **Age 42** |  | |  |  |  |  |  |  |  |
| Household overcrowding∞ | 10171 | | 1.01 | 0.68,1.48 | 0.98 |  | 0.73 | 0.48, 1.12 | 0.15 |
| Unemployed | 10214 | | 1.13 | 0.72,1.75 | 0.60 |  | 0.89 | 0.64, 1.22 | 0.46 |
| Financial difficulties | 10210 | | 1.17 | 0.89,1.54 | 0.27 |  | 1.05 | 0.80, 1.37 | 0.75 |
| Receiving benefits | 10207 | | 0.93 | 0.71,1.23 | 0.62 |  | 0.77 | 0.58, 1.00 | 0.05 |
| Homeless since last sweep | 5407 | | 0.82 | 0.36,1.89 | 0.65 |  | 1.11 | 0.53, 2.33 | 0.78 |
| No car | 8847 | | 0.98 | 0.60,1.58 | 0.92 |  | 1.72 | 1.01, 2.90 | 0.05 |
| Rents from LA or housing association | 10172 | | 0.95 | 0.61,1.50 | 0.83 |  | 1.25 | 0.89, 1.75 | 0.21 |
| **Age 44/ 45** |  | |  |  |  |  |  |  |  |
| Job insecurity | 6971 | | 0.96 | 0.64,1.43 | 0.83 |  | 0.95 | 0.58, 1.56 | 0.84 |
| Owns a car | 7847 | | 1.05 | 0.56,1.96 | 0.89 |  | 0.86 | 0.49, 1.50 | 0.59 |
| Not enough money for food or clothing | 7839 | | 0.74 | 0.51,1.08 | 0.12 |  | 0.88 | 0.62, 1.24 | 0.46 |
| Difficulties paying bills | 7838 | | 0.94 | 0.62,1.44 | 0.79 |  | 1.38 | 0.96, 2.00 | 0.09 |
| 1+ stressful life events in the last 6 months | 7839 | | 0.90 | 0.68,1.21 | 0.49 |  | 1.33 | 0.98, 1.81 | 0.07 |
| **Social support and marital status** | | | | | | | | | |
| Practical social support (medium/ high vs. low)^3^ age 33 | 10224 | | 0.88 | 0.65,1.19 | 0.40 |  | 0.77 | 0.55, 1.07 | 0.12 |
| Emotional social support (medium/ high vs. low)^3^ age 33 | 10224 | | 0.80 | 0.61,1.06 | 0.12 |  | 0.83 | 0.58, 1.19 | 0.31 |
| Social support^4^, age 42 | 10209 | | 1.16 | 0.67,2.02 | 0.59 |  | 1.02 | 0.41, 2.53 | 0.97 |
| Negative support^5^, age 44/ 45 | 7576 | | 0.91 | 0.67,1.23 | 0.54 |  | 0.89 | 0.66, 1.20 | 0.44 |
| Practical support^5^, age 44/ 45 | 7672 | | 0.98 | 0.72,1.35 | 0.92 |  | 1.12 | 0.82, 1.52 | 0.47 |
| Confiding & emotional support^5^ , age 44/ 45 | 7585 | | 1.00 | 0.73,1.36 | 0.98 |  | 1.00 | 0.73, 1.37 | 0.99 |
| Single/ separated/ divorced, age 23 | 11068 | | 1.09 | 0.85,1.41 | 0.49 |  | 1.36 | 1.06, 1.75 | 0.02 |
| Single/ separated/ divorced, age 33 | 9821 | | 1.17 | 0.89,1.55 | 0.26 |  | 1.34 | 1.02, 1.76 | 0.04 |
| Single/ separated/ divorced, age 42 | 10216 | | 1.17 | 0.89,1.54 | 0.27 |  | 1.33 | 1.03, 1.74 | 0.03 |
| **Cohort member psychological health (adulthood)** | | | | | | | | | |
| Adult psychological distress at least once^6^ , age 23, 33 | 8606 | | 1.12 | 0.82,1.53 | 0.49 |  | 1.12 | 0.82, 1.53 | 0.49 |
| Mid-life common mental disorders, age 44/ 45 | 8410 | | 1.07 | 0.66,1.73 | 0.79 |  | 1.40 | 0.98, 1.99 | 0.06 |
| ***Key:*** |  | |  |  |  |  |  |  |  |
| **; **Recalled at mid-life, age 44/ 45; ∞ Household over-crowding: >1 persons/ room vs <=1 person/ room; ‡ p<0.05 for interactions by gender* | | | | | | | | | |
| *^1^No sole access to indoor bathroom, toilet or hot water at least once, age 7, 11 or 16* | | | | | | | | | |
| *^2^At least one of: housing, finances, physical illness/ disability, mental illness, learning disabilities, death of either parent, divorce/ separation, domestic tension, in-law conflicts, unemployment, alcoholism, or any ‘other serious difficulties affecting child’s development’ at age 7* | | | | | | | | | |
| *^3^Emotional and practical social support provided by personal sources and/ or organisational sources, assessed with hypothetical situations* | | | | | | | | | |
| *^4^Does the respondent have someone they could turn to for support* | | | | | | | | | |
| *^5^Confiding, practical and negative support provided from closest nominated person. Assessed through Close Person's Questionnaire.*  *^6^Scores of 8 or more on the Rutter Malaise Inventory* | | | | | | | | | |

**ONLINE REPOSITORY MATERIAL**

**Supplementary table 3**

| **Associations of life-course variables with health-related behaviours at 44/ 45 in the full sample. Univariate analyses, all models have been adjusted for gender** | | | | | | | | | | | | | | | | | |
| --- | --- | --- | --- | --- | --- | --- | --- | --- | --- | --- | --- | --- | --- | --- | --- | --- | --- |
| ***­­*** | ***Binge alcohol use*** | | |  | **Hazardous alcohol use**  **(≥*8 on AUDIT)*** | | | | |  | | ***Smoking*** | | | | | |
|  | **OR** | **95% CI** | **p value** |  | **OR** | **95% CI** | **p value** | | |  | | **OR** | | **95% CI** | | | **p value** |
| ***CHILDHOOD (AGE 7, 11, 16)*** |  |  |  |  |  |  | |  | |  | |  | |  | |  | |
| **Material and social adversity indicators** | | | | | | | | | | | | | | | | | |
| Household overcrowding (at least once at age 7, 11 or 16) | 1.29 | 1.16,1.43 | p<0.001 |  | 1.22 | 1.09,1.36 | | p<0.001 | |  | | 1.37 | | 1.25,1.50 | | p<0.001 | |
| Restricted access to basic household amenities^1^ at age 7, 11 or 16 | 1.11 | 0.98,1.27 | 0.09 |  | 1.21 | 1.06,1.38 | | p<0.001 | |  | | 1.12 | | 1.01,1.25 | | 0.04 | |
| Family financial difficulties, age 7, 11 or 16 | 1.12 | 0.98,1.28 | 0.10 |  | 1.14 | 0.99,1.32 | | 0.06 | |  | | 1.53 | | 1.35,1.72 | | p<0.001 | |
| Family difficulties^2^, assessed by health visitor, age 7 | 1.12 | 0.97,1.29 | 0.12 |  | 1.12 | 0.97,1.29 | | 0.13 | |  | | 1.39 | | 1.22,1.59 | | p<0.001 | |
| Recalled at 44/ 45: 'I grew up in poverty or financial hardship' | 1.12 | 0.98,1.28 | 0.11 |  | 1.23 | 1.07,1.42 | | p<0.001 | |  | | 1.29 | | 1.15,1.46 | | p<0.001 | |
| **Parental health and health-related behaviours** | | | | | | | | | | | | | | | | | |
| Either parent smoked, age 16 | 1.68 | 1.49,1.89 | p<0.001 |  | 1.42 | 1.24,1.63 | p<0.001 | |  | | 1.53 | | 1.37,1.70 | | p<0.001 | | |
| Either parent had alcohol problems, recalled age 44/ 45 | 1.51 | 1.31,1.75 | p<0.001 |  | 1.89 | 1.64,2.19 | p<0.001 | |  | | 1.52 | | 1.33,1.73 | | p<0.001 | | |
| Either parent had emotional/ nervous trouble, recalled age 44/ 45 | 1.05 | 0.94,1.18 | 0.36 |  | 1.35 | 1.20,1.52 | p<0.001 | |  | | 1.16 | | 1.05,1.28 | | p<0.001 | | |
| **Cohort member psychological health (childhood)** | | | | | | | | | | | | | | | | | |
| Childhood internalising problems at least once (age 7, 11, 16) | 0.80 | 0.71,0.90 | p<0.001 |  | 0.92 | 0.81,1.04 | 0.18 | |  | | 1.20 | | 1.08,1.34 | | p<0.001 | | |
| Childhood externalising problems at least once (age 7, 11, 16) | 1.30 | 1.13,1.48 | p<0.001 |  | 1.32 | 1.16,1.51 | p<0.001 | |  | | 2.52 | | 2.23,2.85 | | p<0.001 | | |
| ***ADULTHOOD*** | | | | | | | | | | | | | | | | | |
| **Material and social adversity indicators** | | | | | | | | | | | | | | | | | |
| **Age 23** |  |  |  |  |  |  |  | |  | |  | |  | |  | | |
| Unemployed | 1.27 | 1.04,1.56 | 0.02 |  | 1.54 | 1.27,1.87 | p<0.001 | |  | | 1.85 | | 1.54,2.22 | | p<0.001 | | |
| In receipt of benefits | 1.35 | 1.21,1.52 | p<0.001 |  | 1.29 | 1.14,1.45 | p<0.001 | |  | | 1.95 | | 1.76,2.15 | | p<0.001 | | |
| Homeless since last sweep | 1.15 | 0.92,1.45 | 0.21 |  | 1.61 | 1.28,2.02 | p<0.001 | |  | | 2.12 | | 1.71,2.62 | | p<0.001 | | |
| Council house resident | 1.42 | 1.21,1.67 | p<0.001 |  | 1.37 | 1.16,1.62 | p<0.001 | |  | | 2.05 | | 1.77,2.37 | | p<0.001 | | |
| Shared/ no use of indoor bath or shower | 0.88 | 0.68,1.14 | 0.33 |  | 0.86 | 0.64,1.14 | 0.29 | |  | | 1.26 | | 0.99,1.59 | | 0.06 | | |
| Sole access to indoor toilet | 0.96 | 0.77,1.20 | 0.73 |  | 0.98 | 0.77,1.23 | 0.83 | |  | | 1.13 | | 0.93,1.37 | | 0.23 | | |
| Household overcrowding, (>1 persons/ room )age 23 | 1.70 | 1.17,2.48 | 0.01 |  | 1.23 | 0.87,1.74 | 0.24 | |  | | 1.45 | | 1.08,1.94 | | 0.01 | | |
| **Age 33** |  |  |  |  |  |  |  | |  | |  | |  | |  | | |
| Qualifications vs none | 0.72 | 0.60,0.87 | p<0.001 |  | 0.77 | 0.64,0.92 | p<0.001 | |  | | 0.39 | | 0.33,0.46 | | p<0.001 | | |
| Has central heating | 0.96 | 0.83,1.11 | 0.55 |  | 0.82 | 0.71,0.95 | 0.01 | |  | | 0.64 | | 0.56,0.73 | | p<0.001 | | |
| Lives in social housing | 1.44 | 1.23,1.69 | p<0.001 |  | 1.29 | 1.10,1.51 | p<0.001 | |  | | 2.76 | | 2.38,3.21 | | p<0.001 | | |
| Damp in house | 1.12 | 0.95,1.30 | 0.17 |  | 1.09 | 0.92,1.29 | 0.31 | |  | | 1.31 | | 1.14,1.50 | | p<0.001 | | |
| No access to telephone | 1.05 | 0.84,1.32 | 0.67 |  | 1.34 | 1.07,1.69 | 0.01 | |  | | 2.56 | | 2.04,3.20 | | p<0.001 | | |
| In arrears with bills | 1.69 | 1.17,2.42 | p<0.001 |  | 1.79 | 1.27,2.52 | p<0.001 | |  | | 3.31 | | 2.33,4.70 | | p<0.001 | | |
| Access to household amenities | 1.03 | 0.63,1.68 | 0.91 |  | 1.40 | 0.87,2.26 | 0.16 | |  | | 2.18 | | 1.35,3.51 | | p<0.001 | | |
| Household overcrowding (>1 persons/ room) | 1.26 | 1.07,1.48 | 0.01 |  | 1.10 | 0.93,1.31 | 0.27 | |  | | 1.69 | | 1.45,1.96 | | p<0.001 | | |
| Unemployed | 1.07 | 0.83,1.39 | 0.59 |  | 1.54 | 1.21,1.96 | p<0.001 | |  | | 2.27 | | 1.77,2.91 | | p<0.001 | | |
| **Age 42** |  |  |  |  |  |  |  | |  | |  | |  | |  | | |
| Household overcrowding, (>1 persons/ room) | 1.01 | 0.85,1.20 | 0.90 |  | 0.76 | 0.64,0.89 | p<0.001 | |  | | 1.06 | | 0.92,1.23 | | 0.42 | | |
| Unemployed | 0.78 | 0.67,0.89 | p<0.001 |  | 1.22 | 1.03,1.44 | 0.02 | |  | | 1.48 | | 1.30,1.69 | | p<0.001 | | |
| Financial difficulties | 1.04 | 0.93,1.16 | 0.48 |  | 1.16 | 1.03,1.30 | 0.01 | |  | | 1.79 | | 1.62,1.97 | | p<0.001 | | |
| Receiving benefits | 0.94 | 0.84,1.05 | 0.25 |  | 0.79 | 0.71,0.88 | p<0.001 | |  | | 0.94 | | 0.85,1.03 | | 0.19 | | |
| Homeless since last sweep | 1.34 | 1.00,1.79 | 0.05 |  | 1.44 | 1.10,1.90 | 0.01 | |  | | 1.75 | | 1.33,2.29 | | p<0.001 | | |
| No car | 1.43 | 1.13,1.81 | p<0.001 |  | 1.50 | 1.22,1.83 | p<0.001 | |  | | 1.32 | | 1.09,1.59 | | p<0.001 | | |
| Rents from LA or housing association | 1.44 | 1.22,1.71 | p<0.001 |  | 1.43 | 1.20,1.70 | p<0.001 | |  | | 3.12 | | 2.65,3.68 | | p<0.001 | | |
| **Age 44/ 45** |  |  |  |  |  |  |  | |  | |  | |  | |  | | |
| Job insecurity | 1.05 | 0.90,1.22 | 0.56 |  | 1.28 | 1.10,1.49 | p<0.001 | |  | | 1.22 | | 1.07,1.39 | | p<0.001 | | |
| Does not own a car | 1.32 | 1.06,1.65 | 0.01 |  | 2.08 | 1.69,2.57 | p<0.001 | |  | | 1.82 | | 1.50,2.22 | | p<0.001 | | |
| Money for food or clothing | 1.11 | 0.99,1.25 | 0.09 |  | 1.21 | 1.07,1.37 | p<0.001 | |  | | 1.61 | | 1.44,1.79 | | p<0.001 | | |
| Difficulties paying bills | 1.16 | 1.00,1.34 | 0.05 |  | 1.32 | 1.14,1.54 | p<0.001 | |  | | 1.76 | | 1.55,2.01 | | p<0.001 | | |
| 1+ stressful life events in the past 6 months | 1.17 | 1.06,1.30 | p<0.001 |  | 1.25 | 1.12,1.40 | p<0.001 | |  | | 1.05 | | 0.96,1.15 | | 0.28 | | |
| **Social support and marital status** | | | | | | | | | | | | | | | | | |
| Practical social support (medium/ high versus low)^3^, 33 | 0.97 | 0.84,1.12 | 0.67 |  | 0.88 | 0.76,1.02 | 0.10 | |  | | 0.74 | | 0.65,0.85 | | p<0.001 | | |
| Emotional social support (medium/ high vs low)^3^, 33 | 0.96 | 0.83,1.11 | 0.57 |  | 0.89 | 0.77,1.03 | 0.11 | |  | | 0.81 | | 0.71,0.92 | | p<0.001 | | |
| Social support^4^, 42 | 0.72 | 0.54,0.96 | 0.03 |  | 0.89 | 0.67,1.19 | 0.43 | |  | | 1.09 | | 0.85,1.40 | | 0.49 | | |
| Negative social support^5^, 44/ 45 | 0.98 | 0.89,1.08 | 0.68 |  | 1.16 | 1.04,1.29 | 0.01 | |  | | 1.04 | | 0.95,1.14 | | 0.39 | | |
| Practical social support^5^, 44/ 45 | 0.91 | 0.82,1.01 | 0.07 |  | 0.87 | 0.78,0.97 | 0.02 | |  | | 0.98 | | 0.89,1.07 | | 0.60 | | |
| Confiding and emotional social support^5^, 44/ 45 | 0.92 | 0.83,1.03 | 0.14 |  | 0.84 | 0.75,0.94 | p<0.001 | |  | | 0.92 | | 0.83,1.01 | | 0.07 | | |
| Single/ separated/ divorced versus married, age 23 | 1.01 | 0.91,1.12 | 0.87 |  | 1.10 | 0.98,1.23 | 0.10 | |  | | 0.88 | | 0.80,0.96 | | 0.01 | | |
| Single/ separated/ divorced versus married, age 33 | 1.05 | 0.93,1.17 | 0.44 |  | 1.34 | 1.19,1.51 | p<0.001 | |  | | 1.30 | | 1.18,1.44 | | p<0.001 | | |
| Single/ separated/ divorced versus married, age 42 | 1.18 | 1.06,1.32 | p<0.001 |  | 1.51 | 1.35,1.69 | p<0.001 | |  | | 1.36 | | 1.23,1.49 | | p<0.001 | | |
| **Cohort member psychological health (adulthood)** | | | | | | | | | | | | | | | | | |
| Adult psychological distress at least once (age 23, 33, 42)^6^ | 1.20 | 1.05,1.37 | 0.01 |  | 1.81 | 1.57,2.08 | p<0.001 | |  | | 1.90 | | 1.68,2.15 | | p<0.001 | | |
| Mid-life common mental disorders (age 44/ 45) | 1.17 | 1.00,1.36 | 0.05 |  | 1.71 | 1.46,2.00 | p<0.001 | |  | | 1.43 | | 1.25,1.65 | | p<0.001 | | |
| ***Key*** |  |  |  |  |  |  |  | |  | |  | |  | |  | | |
| *^1^ No sole access to indoor bathroom, toilet or hot water at least once, age 7, 11 or 16* | | | | | | | | | | | | | | | | | |
| *^2^ At least one of: housing, finances, physical illness/ disability, mental illness, learning disabilities, death of either parent, divorce/ separation, domestic tension, in-law conflicts, unemployment, alcoholism, or any ‘other serious difficulties affecting child’s development’ at age 7* | | | | | | | | | | | | | | | | | |
| *^3^ Emotional and practical social support provided by personal sources and/ or organisational sources, assessed with hypothetical situations* | | | | | | | | | | | | | | | | | |
| *^4^ Does the respondent have someone they could turn to for support* | | | | | | | | | | | | | | | | | |
| *^5^ Assessed through Close Person's Questionnaire.*  *^6^ Scores of 8 or more on the Rutter Malaise Inventory* | | | | | | | | | | | | | | | | | |

**Figure 1 (in colour):** Plot of relative odds of being a smoker/ ex-smoker vs. non-smoker in second generation Irish men and women relative to men and women in the rest of the cohort

*Key*

*Black circles indicate ORs in second generation Irish men relative to men in the non-Irish reference group; Red triangles indicate ORs in second generation Irish women relative to women in the non-Irish reference group. Horizontal lines indicate 95% CIs. Estimates falling on the vertical line indicate no difference between second generation Irish participants and the non-Irish reference group. *p value for interaction of gender with ethnicity: p=0.02*
